# Physical health monitoring in antipsychotic depot clinic

**DOI:** 10.1192/bjo.2021.836

**Published:** 2021-06-18

**Authors:** Rebecca Davies, Anu Priya, Hardev Bhogal, Adesola Omodara, George Davies, Shweta Mittal

**Affiliations:** 1 Nottinghamshire Healthcare NHS Foundation Trust; 2 Nottingham Medical University

## Abstract

**Aims:**

A service evaluation project to look at if annual bloods, ECG, physical examination, and medical review was completed within the last year for patients attending anti-psychotic depot clinic at Bassetlaw mental health services in Nottinghamshire HealthCare NHS Foundation Trust.

**Method:**

Electronic notes were examined in October 2020 for 25 patients who attend anti-psychotic depot clinic to ascertain if medical review and physical examination had been completed along with annual bloods and ECG.

**Result:**

Out of 25 patients attending depot clinic in 2020 at Bassetlaw Hospital, 21 had all their blood tests done, 1 patient had refused bloods and 2 patients did not have blood tests done. ECG was completed for 3 patients at Bassetlaw hospital and 8 patients had it requested from primary care with 2 patients refusing to have ECG done. For 12 patients there was no evidence of ECG being requested or completed. 8 patients had physical examination completed and rest 17 patients did not have the physical examination completed including due to refusal. Out of 25, only 14 patients had a medical review conducted.

**Conclusion:**

Patients who attend depot clinic may have an allocated community psychiatric nurse (CPN) or get reviewed by medics in outpatient clinics and would usually have their blood tests, physical health examination and ECGs requested and monitored by them. Patients who do not have any allocated CPN or medic tend to miss out on blood tests and ECG. General Practitioners are expected to complete physical health checks for patients who do not have CPN or regular outpatient review. The results of these investigations may not always be received in depot clinic, hence there is no documentation on electronic RIO system. When these patients disengage from the depot clinic, it is often very difficult to track them. As a follow-up from this service evaluation, all depot clinic patients will be allocated a key worker/CPN. This will ensure that they have a responsible person to facilitate annual checks. This will be reviewed in a years' time to evaluate the effectiveness of this intervention.

